# Surface Activation of Titanium Dental Implants by Using UVC-LED Irradiation

**DOI:** 10.3390/ijms22052597

**Published:** 2021-03-05

**Authors:** Nagore Arroyo-Lamas, Iciar Arteagoitia, Unai Ugalde

**Affiliations:** 1 Medicine and Surgery Program, PhD School, University of the Basque Country UPV/EHU, Leioa, 48940 Bizkaia, Spain; narroyo005@ikasle.ehu.eus; 2 Maxillofacial Group, Stomatology Department, BioCruces Health Research Institute, University of the Basque Country UPV/EHU, Leioa, 48940 Bizkaia, Spain; 3 APERT Research Group, Department of Electronic Technology, University of the Basque Country, Bilbao, 48013 Bizkaia, Spain; unai.ugalde@ehu.eus

**Keywords:** titanium, dental implants, ultraviolet rays, hydrocarbons, decontamination, microscopy, electron, scanning, photoelectron spectroscopy

## Abstract

Organic contaminants significantly limit the bioactivity of titanium implants, resulting in the degradation known as the ageing of titanium. To reactivate the surfaces, they can be photofunctionalized, i.e., irradiated with C-range ultraviolet (UVC) light. This descriptive in vitro study compares the effectiveness of novel light-emitting diode (LED) technology to remove contaminant hydrocarbons from three different commercially available titanium dental implants: THD, TiUnite, and SLA. The surface topography and morphology were characterized by scanning electron microscopy (SEM). The chemical compositions were analyzed by X-ray photoelectron spectroscopy (XPS), before and after the lighting treatment, by a pair of closely placed UVC (λ = 278 nm) and LED devices for 24 h. SEM analysis showed morphological differences at the macro- and micro-scopic level. XPS analysis showed a remarkable reduction in the carbon contents after the UVC treatment: from 25.6 to 19.5 C at. % (carbon atomic concentration) in the THD; from 30.2 to 20.2 C at. % in the TiUnite; from 26.1 to 19.2 C at. % in the SLA surface. Simultaneously, the concentration of oxygen and titanium increased. Therefore, LED-based UVC irradiation decontaminated titanium surfaces and improved the chemical features of them, regardless of the kind of surface.

## 1. Introduction

Most dental implants are made of titanium (Ti) due to its high corrosion resistance, low modulus of elasticity, good fatigue strength, and non-cytotoxic features, resulting in a favorable biocompatible material with excellent osseointegration ability, defined as a direct, strong, stable, and durable connection in function between artificial implants and bone [1,2]. Nevertheless, it is not sufficient and, despite their high long-term predictability [3,4], several external factors limit the osseointegration of Ti-based implants, which may cause implant failure, such as the presence of poor bone quality and quantity, bone defects, systematic diseases, or bacterial infections [5,6]. In order to minimize this, new bioactive surface treatments for Ti-based implants are currently being developed, which foster bone–implant interactions and reduce bacterial attachment, providing high long-term success rates [7,8]. Thereby, microscopic modifications in topography and chemistry in titanium implants have been implemented to produce osteoconductive and antimicrobial implants [9,10,11].

In this context, the inevitable presence of carbon-based organic impurities on Ti surfaces is increasingly being regarded as one of the major factors causing biological degradation, often referred to as the ageing of titanium [12]. Indeed, not only does this accumulation of carbon-based contaminants, e.g., polycarbonlys and hydrocarbons, hinder the attachment and proliferation of proteins and cells but it also facilitates bacteria adhesion and hydrophobicity, since cell and bacteria–oxide layer interaction occurs at the atomic level [13,14,15]. Likewise, several investigations have reported changes in the presence of chemical elements, especially carbon contents, associated with the different surface treatments to which the titanium dental implants are subjected [16,17].

It is well known that ultraviolet light, particularly in the C-range (UVC), successfully eliminates carbon-based contaminants from Ti surfaces. Such organic compounds are then broken down, either directly by the photogenerated holes or indirectly by hydroxyl radicals resulting from water decomposition [18,19]. When this novel technique is applied to Ti-based dental implants, it is called photofunctionalization. It significantly reactivates the surfaces and improves the osseointegration by promoting faster osteoblast attachment, proliferation, and differentiation; high implant stability quotient (ISQ); bone–implant contact (BIC); low surface free-energy (superhydrophilicity); and a reduction of biofilm formation [20,21,22,23,24,25]. Furthermore, the removal of hydrocarbons from the Ti surfaces, resulting in the exposure of Ti^4+^ sites, may enhance the interaction between Ti-based material and biological cells, which are electronegatively charged, promoting the bone formation process [12]. In addition, the albumin, known as a major blood plasma protein that regulates cellular proliferation of osteoblasts, is adsorbed in the first steps of osseointegration and is influenced by carbon contents, covalent bonds, and electrostatic charge [15]. The adsorption of other human blood proteins and cell attachment are also strongly correlated with UV dose and carbon percentage, since the UV light treatment considerably increases the biochemical interlocking between material and matrix proteins and bone cells [26].

Until recently, UV light has been almost universally produced by using mercury (Hg)-vapor lamps. This is basically the same technology used in fluorescent tubes and compact bulbs for general lighting purposes [27]. Indeed, photofunctionalization has been no exception, but because of the upcoming restrictions on the manufacture and trade of Hg-based products imposed by the Minamata Convention [28], other options such as LED (light-Emitting diode)-based irradiators are being proposed as alternative sources. The use of these devices is considered a novel approach, compared to the use of those based on Hg vapor. Hence, these light sources show promising results and demonstrate an effective photofunctionalized alternative to hydrocarbon decontamination [29]. Currently, however, there are no published studies in which this LED-based technology is applied to different types of surfaces.

We hypothesized that UVC light emitted by LED-based sources would successfully remove contaminant hydrocarbons from different titanium surfaces. The aim of the present in vitro study therefore was to compare the effectiveness of LED-based UVC photofunctionalization technology to decontaminate the surface chemistry of three different commercially available titanium dental implants.

## 2. Results

### 2.1. Scanning Electron Microscopy (SEM) Analysis

**Figure 1:**
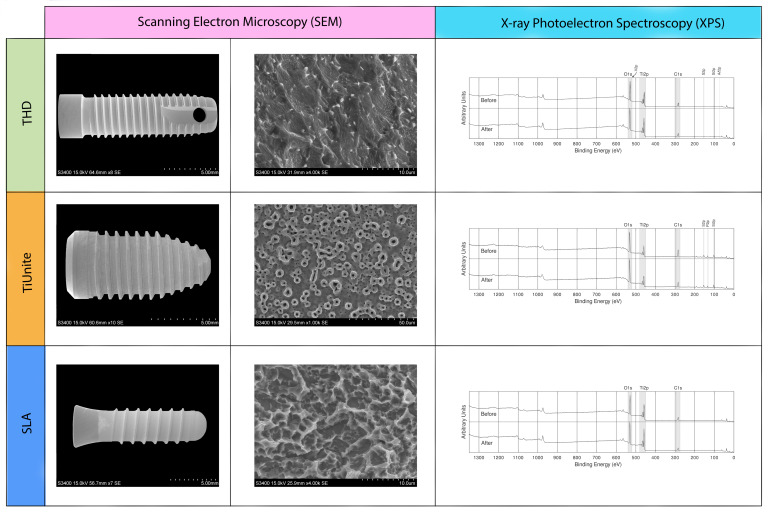
SEM pictures (at the macro- and micro-scopic level, magnifications of 7–10× and 1000×, respectively) and XPS wide scans of Ti dental implants: THD; TiUnite; SLA.

The topographic and morphologic features of the three different titanium dental surfaces were evaluated by SEM (Figure 1). Additionally, macroscopic differences were exhibited and, according to the surface modification methods used, characteristic differences at the microlevel were also revealed. The THD implant presented facets induced by fine etching pits and blasting methods. The TiUnite implant featured a volcano-like microporous structure produced by anodic electrochemical oxidation phenomena. The SLA implant had large dips, pointed dips, and small micropits with cristallographically oriented boundaries, which gave a honeycomb appearance, as a result of acid-etched and sand-blasting treatments.

### 2.2. X-ray Photoelectron Spectroscopy (XPS) Analysis

All of the detected elements were presented in high-resolution spectra of the wide scans (Figure 1) and the relative atom concentrations (at. %) and binding energies of each sample before and after UVC light treatment (Table 1).

The survey spectra of as-removed packaging implants revealed the presence of carbon (C), oxygen (O), and titanium (Ti) on all the implant surfaces. However, other elements such as aluminum (Al), vanadium (V), and silicon (Si) were also observed in THD surface., and TiUnite showed concentrations of silicon (Si) and phosphorus (P). In addition, a little debris of fluoride deposit appeared in THD and SLA regimes.

All samples showed a higher carbon content before lighting treatment, with a wide range from 30.2 to 25.6 at. % (atomic concentration). Likewise, LED-based UVC photofunctionalization notably reduced the concentration of the carbon species, regardless of the type of surface used, from 20.2 to 19.2 at. %. In addition, the decrease in carbon triggered a considerable boost in the atomic concentration of O and Ti.

**Figure 2:**
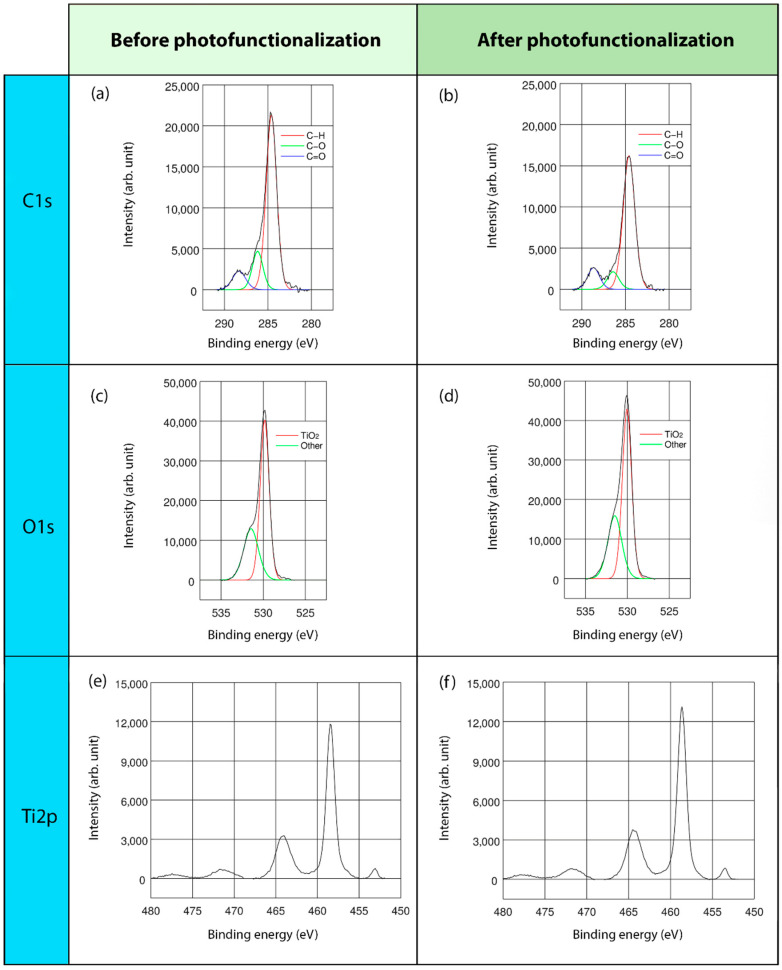
XPS analyses of THD surface: XPS full-range spectra and deconvoluted XPS, with lines analysis and binding energies of C1s, O1s, and Ti2p; before (**a**,**c**,**e**) and after (**b**,**d**,**f**) LED-based UVC photofunctionalization.

**Figure 3:**
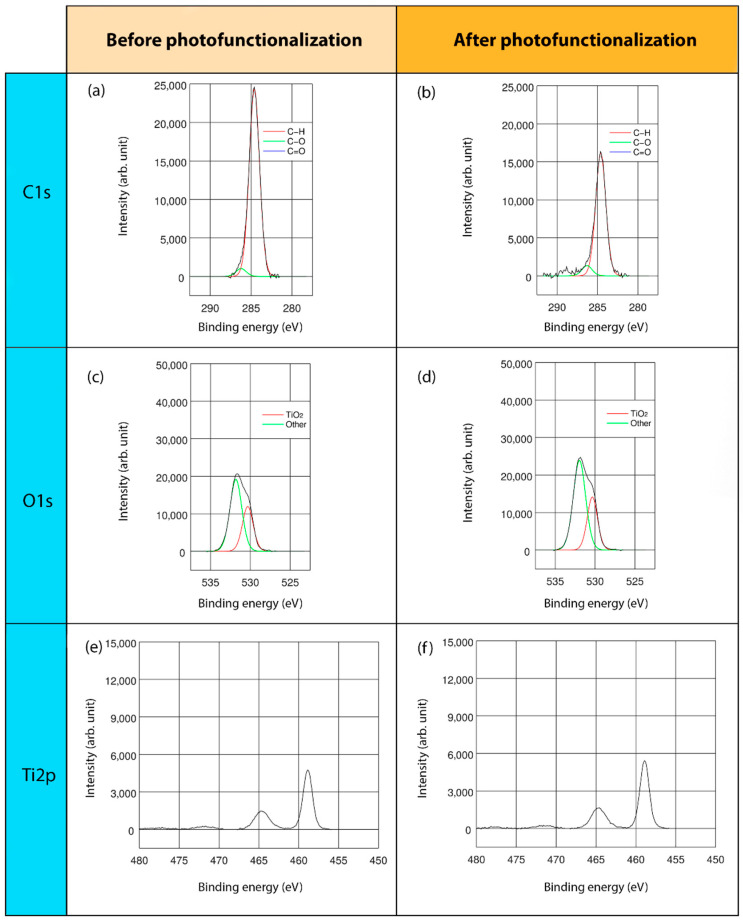
XPS analyses of TiUnite surface: XPS full-range spectra and deconvoluted XPS, with lines analysis and binding energies of C1s, O1s and Ti2p; before (**a**,**c**,**e**) and after (**b**,**d**,**f**) LED-based UVC photofunctionalization.

**Figure 4:**
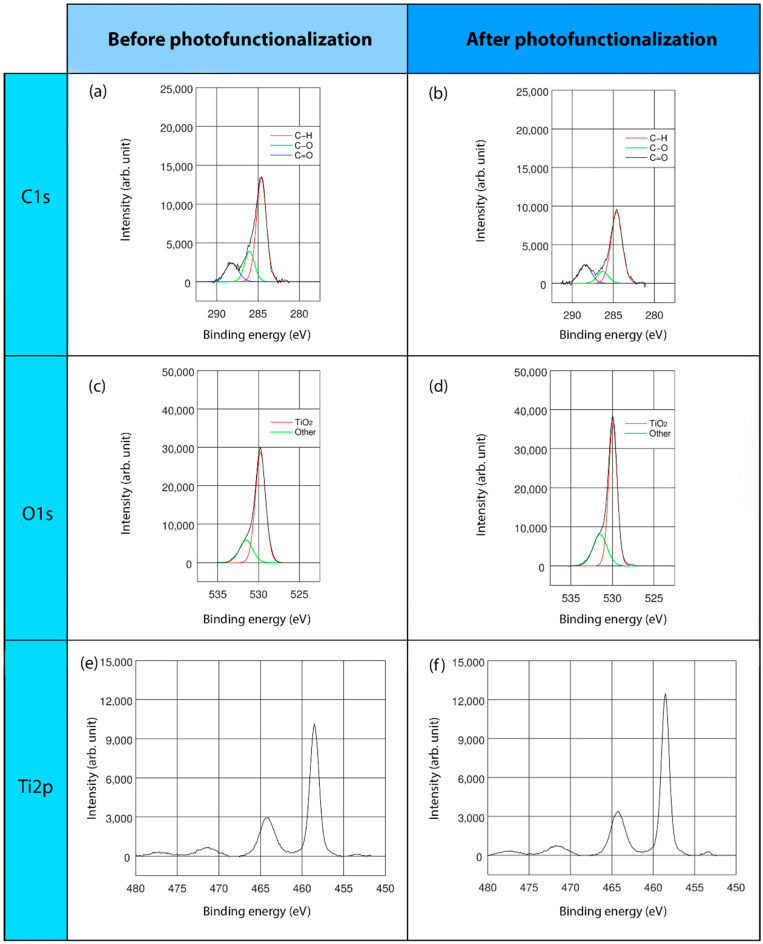
XPS analyses of SLA surface: XPS full-range spectra and deconvoluted XPS, with lines analysis and binding energies of C1s, O1s, and Ti2p; before (**a**,**c**,**e**) and after (**b**,**d**,**f**) LED-based UVC photofunctionalization.

This study mainly concentrated on C, O, and Ti elements, namely, the major elements detected on the surfaces of all the regimes and presented in the stoichiometrical deconvolutions of C1s, O1s, and Ti2p of three surfaces, before and after the lighting treatment (Figure 2, Figure 3 and Figure 4).

In the high-resolution spectral profile of the C1s deconvolution of the three different surface regimes, three energy peaks were found: at 284.6, 286.2, and 288.3 eV. The predominant peak, at 284.6 eV, corresponded to the percentage of hydrocarbons, specifically, C-H bonds. The second and third peaks, represented C-O and C=O bonds, respectively (Figure 2a,b, Figure 3a,b and Figure 4a,b). In particular, TiUnite showed the greatest amount of carbon content. Generally, the hydrocarbon concentration decreased significantly after UVC light irradiation in all of the surfaces.

The O1s core lines consisted of two components (Figure 2c,d, Figure 3c,d and Figure 4c,d). The main peaks close to 529.9–530.0 eV were related to TiO_2_ bonds, whereas the second component corresponded to a vast array of bonds, such as Ti-OH and C=O. Due to the low significant peaks of these components, they could not be accurately identified.

Regarding the Ti double-peak spectra assessment, one component was obtained (Figure 2e,f, Figure 3e,f, and Figure 4e,f). The binding energies at 458.4–458.6 eV were ascribed to TiO_2_ compounds. Furthermore, the metallic form of Ti was also observed at 453.8–453.9 eV in THD and SLA samples.

## 3. Discussion

In the present study, the successful decontamination of hydrocarbons of three different surfaces by using a UVC-LED-based photofunctionalization device was assessed. After 24 h of irradiation, all titanium dental implants showed a significant drop in hydrocarbon indices, associated with a sharp increase in O and Ti concentration. Therefore, UVC LED-based technology is an effective method to overcome the biological aging of titanium, modifying its chemical structure, regardless of the type of surface used and, thereby, reactivating the implant surface.

In this investigation, some of the most common titanium surfaces widely used (acid-etched, anodized, and sandblasting), were chosen, but there is a wide range of possibilities available on the market. In this context, SEM characterization revealed morphological differences at macro- and micro-scopic levels. In addition, in the XPS measurements, it is evident that the major elements, i.e., C, O, and Ti, were always the same in each sample, irrespective of the kind of surface employed. Indeed, similar results are also reported in the literature [16,30]. 

Several studies on the surface chemistry underline more complicated compositions involving other organic species, such as fluoride (F), nitrogen (N), or phosphorus (P), correlated to the surface treatment methods applied [16], e.g., the use of strong acids for etching, such as hydrochloric or nitric acids. The presence of aluminum (Al) and vanadium (V) is associated with the most common titanium alloy (Ti-6Al-4V) employed in dental implant production [31]. Additionally, silicon (Si) and sulphur (S) contents, organosilicon and sulphur compounds, would be produced during the cleaning techniques or lubricant residues in the manufacturing process [16].

Unfortunately, Ti surfaces unavoidably attract ubiquitous hydrocarbons from the atmosphere, which justifies the high presence of carbon content in all the samples. Although the hydrocarbons’ percentage of all specimens was notably reduced after LED-based UVC irradiation, the TiUnite surface obtained considerably more effective results. Indeed, it resulted in a reduction from 28.93 to 17.81 at. %, as the C1s spectra deconvolution at BE = 284.6 eV shows. Likewise, it was observed that anodically oxidized surfaces contained notably more hydrocarbons than the others before the lighting treatment [32].

Generally, acid-etched implants present lower hydrocarbon concentrations than machined surfaces [30]. Furthermore, compared to 4-week-old acid-etched Ti surfaces, the newly manufactured implants present lower carbon concentrations, showing high rates of oxygen-containing hydrocarbons on old surfaces that are not observed in newly manufactured surfaces [33]. In this regard, both the amount of adsorbed albumin and the number of attached osteoblasts show significant linear inverse correlations to carbon content percentage. Thus, the higher the percentages of carbon compounds, the lower the amounts of albumin and the number of cells attached to the titanium surface [26]. Moreover, the hydrocarbon-contaminated surfaces exhibit rounded osteoblasts, with a suppression in the cytoskeleton formation and a late cellular proliferation [34]. Decontaminated surfaces have proven a considerable promotion of cellular phenotype [20,35]. Indeed, an important aspect to consider is the way in which the interactions occurred and how organic molecules, such as proteins or peptides, bind to the TiO_2_ surface. Specifically, these interactions might occur at an electrostatic level by two different mechanisms. One way may be between bonds formed by positively charged amino acid groups (e.g., –NH^3+^) and the TiO_2_ surface, which is negatively charged. The alternative way may be between positively charged Ca^++^ bridges, which have been previously bonded to a negatively charged TiO_2_ surface and negatively charged amino acid groups (–COO^−^) [9].

It must be highlighted that it was not the purpose of this investigation to provide surface composition data of commercially available titanium dental implants. This information is already reported in the scientific literature [16].

UV irradiation can be subdivided into three categories based on its wavelengths: UVC or short wave (λ = 200–280 nm), UVB or medium wave (λ = 280–320 nm), and UVA or large wave (λ = 320–400 nm). Although the three wavelengths have been analyzed [36,37,38], by far the most studied have employed UVA and UVC light sources. Nevertheless, given that both UVA and UVC trigger wettability changes (i.e., from hydrophobic to superhydrophilic surfaces), only UVC shows better decontamination rates of hydrocarbons, bacteria, and biological effects. Therefore, UVC light is likely an efficient method of photofunctionalization [12,15,20,24,36]. Moreover, neither UVA nor UVC light irradiation cause topographic or morphologic changes on Ti implant surfaces [10,39].

Previous in vitro and in vivo studies employed custom-photofunctionalized disks and cylinders [10,11,15,40]. Notwithstanding, little research has been conducted to determine the effects of UVC photofunctionalization on commercially available Ti dental implants, specifically, to clarify the chemical modifications of Ti implants and the possible influences on the osseointegration process. Therefore, we conducted the present study with high clinical implications to understand the photo-induced mechanism on implant surfaces.

Our findings show a successful response to remove hydrocarbons from a vast array of marketed implant surfaces. Roy et al. obtained similar outcomes [41]. Additionally, an in vitro implant study suggested the influences of photofunctionalization on the modulation of the early inflammatory human response, which positively enhance the osseointegration [42]. Meanwhile, the retrospective clinical studies report an increase in ISQ and marginal bone level rates after UV photofunctionalization, increasing the success rates up to 97.6% for 2.5 years of loading [22,43,44]. However, long-term clinical research needs to be conducted.

Nonetheless, the main limitation of the present research is that it is an in vitro characterization. In light of such encouraging and positive results, in vivo implications must be confirmed in further clinical studies to determine the photofunctionalization influence on osseointegration.

## 4. Materials and Methods

### 4.1. Sample Selection

Three original screw-type, commercially available Ti dental implants with different surfaces were investigated in this descriptive in vitro study: THD (16 × 3.8 mm, Sterioss, Anaheim, CA, USA), TiUnite (5 × 10 mm, Nobel Biocare, Gothenburg, Sweden), and SLA (12 × 4.1 mm, Institut Straumann AG, Basel, Switzerland). The length and diameter of the implants were established according to the description of the manufacturers.

The purpose was to constitute a good share of the worldwide marketed titanium dental implant surfaces. Therefore, for each group, commonly sold surfaces were selected, divided into 3 groups, according to their surface finish: acid-etched and sandblasted (THD); anodically oxidized (TiUnite); or acid-etched, large-grit, and sandblasted (SLA).

### 4.2. Ultraviolet Irradiation Treatment

The specimens were UVC-irradiated under ambient conditions for 24 h in a custom-designed LED-based device (λ = 278 nm) (LEDs: LEUVA66B00HF00; LG Innotek, Seoul, South Korea) with a light power source of 2 mW [25]. The samples rotated vertically in the device, which had aluminum walls, which ensured the UVC light illuminated uniformly on all sample surfaces. The 24 h-period was measured by a digital timer, and the distance between the LEDs and the implants was 2 cm.

### 4.3. Surface Analysis

Scanning electron microscopy (SEM) and X-ray photoelectron spectroscopy (XPS) were used to characterize the surface topography, morphology, and chemical composition of the samples.

#### 4.3.1. Scanning Electron Microscopy (SEM)

The surface topography and morphology of the three Ti implants were acquired by scanning electron microscopy (SEM; S-3400N, Hitachi, Tokyo, Japan) under vacuum. Sterile forceps were used to locate them in a SEM and avoid contamination. Implants were analyzed without the addition of a conductive coating, with acceleration voltage of 15 kV. To verify that the surfaces were different at the macro- and micro-scopic levels, magnifications of 7–10× and 1000×, respectively, were used.

#### 4.3.2. X-ray Photoelectron Spectroscopy (XPS)

For chemical composition assessment, after being removed from their original packaging, all samples were placed on the metal deck of the XPS chamber equipment and introduced into it in order to analyze the surface chemistry prior to the UV treatment. For each sample, three different evaluation points on the implant surface were analysed to ensure homogeneity:

- THD: between threads 4 and 5, 6 and 7, and 19 and 20 of the implant;
- TiUnite: between threads 2 and 3, 4 and 5, and 9 and 10 of the implant;
- SLA: between threads 2 and 3, 4 and 5, and 7 and 8 of the implant.

The measurements of the XPS were conducted in equipment (SPECS System, Berlin, Germany) with a Phoibos analyzer 150 1D-DLD and monochromatic Al Kα (1486.7 eV) X-ray source. Vacuum pressure of 5 × 10^−5^ mbar was used to analyze the spectral data at a 90° exit angle and 1 × 3 mm of the measured area. First, a wide scan was carried out to determine the elements present on the surface (step energy 1 eV, dwell time 0.1 s, pass energy 80 eV). A detailed narrow scan was performed next, concentrating on the major elements detected (step energy 0.1 eV, dwell time 0.1 s, pass energy 30 eV). The C1s peak (hydrocarbons C-C and C-H) was used to calibrate the binding energies.

As described in Section 4.2, the specimens were then UVC-treated, i.e., photofunctionalized, and the XPS analyses were repeated exactly as described above, so the same information was available for the before and after UVC light-treated samples for each different surface.

### 4.4. Statistical Analyses

CasaXPS 2.3.16 software (Casa Software Ltd.; Teignmouth, Devon, UK) was employed to adjust the XPS spectra, which models the Gauss–Lorentzian contributions, after a background subtraction (Shirley). Deconvolutions of the detected elements and a descriptive assessment were made.

## 5. Conclusions

The outcomes of this descriptive study suggest that decontamination of titanium surfaces occurs after LED-based UVC photofunctionalization (λ = 278 nm), decreasing carbon compounds regardless of the kind of surface used. Therefore, this may allow improving the chemical characteristics of titanium dental implants, thereby reactivating the surface features.

## Figures and Tables

**Table 1:** Atom concentration rate (at. %) and binding energies (BE) of elements before and after LED-based UVC light irradiation. These are the results of the central point, between the 6th and 7th thread in the THD, and 4th and 5th thread in the TiUnite and SLA, since the data of the other measurements do not differ.

Elements	BE	THD		TiUnite		SLA	
		Before	After	Before	After	Before	After
		at. %	at. %	at. %	at. %	at. %	at. %
C	284.6–288.3	25.6	19.5	30.2	20.2	26.1	19.2
O	529.8–531.5	50.5	54.1	44.5	51.3	50.6	56.5
Ti	453.7–477.3	16.5	18.0	9.0	9.3	21.2	22.9
F	648.8	0.5	0.6	-	-	0.8 *	-
N	400.3	1.2*	1.3*	-	1.5*	1.3 *	1.4 *
Al	74.0	2.3	2.7	-	-	-	-
Si	102.0	2.8	3.2	11.2	11.8	-	-
S	169.0	-	-	-	1.0*	-	-
V	515.1	0.5 *	0.6 *	-	-	-	-
P	133.3–134.3	-	-	5.1	5.0	-	-

* Spectra close to the noise; at. %, atomic concentration; BE, binding energy.
